# Psychometric Properties of Social Climate in the Classroom Scale for University Students in Chile

**DOI:** 10.3390/bs14111057

**Published:** 2024-11-06

**Authors:** Camila León, Mónica Bravo-Sanzana, Oscar Terán-Mendoza

**Affiliations:** 1 Magister en Psicología, Universidad de La Frontera, Temuco 1145, Chile; c.leon03@ufromail.cl; 2 Núcleo Científico-Tecnológico en Ciencias Sociales y Humanidades, Universidad de La Frontera, Temuco 1145, Chile; 3 Doctorado en Psicología, Universidad de La Frontera, Temuco 1145, Chile; o.teran01@ufromail.cl

**Keywords:** psychometrics, classroom social climate, educational psychology, scale, university students

## Abstract

Background: Classroom social climate is a significant phenomenon within educational contexts; however, it has predominantly been studied among high school students, with limited evidence available at the university level and, consequently, in the psychometric properties of measurement instruments. This study aimed to analyze the psychometric properties of the University Classroom Social Climate Scale (ECSA-U) among Chilean students from the La Araucanía region. Method: 422 students participated, responding to the adapted version of the ECSA-U and the Motivation subscale of the Motivation and Learning Strategies Questionnaire. Exploratory structural equation modeling (ESEM), reliability analyses, and correlation analyses were conducted to provide valid evidence for the Chilean University Classroom Social Climate Scale (ECSA-UCL). Results: The scale demonstrated a three-factor structure with good fit indicators, excellent reliability indices, and significant positive associations between the ECSA-UCL and the Motivation Subscale. Conclusions: The ECSA-UCL has proven valid and reliable for measuring the perception of Classroom Social Climate among Chilean university students, making it suitable for use as a measurement tool in studies or interventions that include this variable.

## 1. Introduction

University is an essential institution in society, pivotal in advancing science, technology, arts, and humanities. It contributes to developing highly skilled professionals, facilitates knowledge creation and application, and fosters innovation and knowledge transfer [1]. In Chile, higher education is considered a fundamental right and includes universities, professional institutes, and technical centers [2]. Over the past 50 years, the governance and structure of this system have been significantly transformed through reforms focused on enhancing equity and quality [3]; these changes respond to critiques of the system’s neoliberal mode [4], a model associated with considerable social unrest in the country [5,6].

To ensure educational quality and equity, higher education institutions are challenged to prioritize students and their learning while fostering inclusion, respect, and human rights—particularly within workplace relationships and campus life [2]. This focus has driven the development of state policies in Chile and research initiatives related to educational coexistence and classroom climate in higher education. These efforts aim to move beyond mere prevention, centering instead on the well-being of the educational community as a whole [7].

From this perspective, academic success or failure, motivation, and phenomena such as higher education dropout rates can be related to several factors, whether economic, socioemotional, or structural [8,9]. However, only a few studies have investigated how course structure or teacher competencies impact these processes [10]. In this context, the Classroom Social Climate (CSC) addresses the internal dynamics of the course’s students, capturing the perceived connection between teachers and students within the classroom, arising from both their interpersonal relationships and the course’s structure or organization, embodying a specific understanding of the crucial elements of this construct [11].

The definition of the Classroom Social Climate (CSC) is an ongoing debate; however, it has been possible to identify three critical elements within the classroom that shape interactions between teachers and students: pedagogical support, socioemotional support, and classroom organization and management [12]. Highlighting the importance of CSC, numerous studies with elementary and secondary students have shown that it can be viewed from various angles [13,14,15,16,17,18,19], touching on everything from teaching methods to overall school management and linked to vital concepts like school harmony and student wellbeing. Additionally, Wang et al. [12] point out that CSC not only influences academic and behavioral outcomes but also affects the socioemotional traits of students.

Despite the valuable contributions that the Classroom Social Climate (CSC) can make to the educational process, research into CSC characteristics, specifically in university students, is scarce. Research in international contexts has identified positive and negative effects of CSC in higher education. Positively, CSC has been shown to bolster self-esteem and a positive self-concept among students. It encourages students to value their achievements and view challenges as opportunities for learning, especially when they perceive support in tackling complex tasks [20]. Studies indicate that CSC enhances understanding of student development, motivation, and phenomena such as persistence or academic dropout and is a helpful tool for assessing the quality of educational environments [21,22].

Conversely, a negative perception of CSC is characterized by a tense and authoritarian atmosphere that affects student participation and collaboration, ultimately diminishing motivation. In addition, a negative perception of CSC may be associated with higher dropout rates [23]. Such an atmosphere might adversely affect students’ self-esteem and their adjustment processes to university life [24]. A discouraging environment within the classroom can restrict student actions, diminish self-concept, personally and academically, and lead to demotivation [25].

In Latin America, research on Classroom Social Climate (CSC) primarily examines variables such as life satisfaction, school coexistence, academic performance, and adverse phenomena like school violence [26,27]. A systematic review of this construct in Latin America and Spain revealed that a negative perception of CSC reduces student motivation and engagement, leading to challenges in managing emotions and sense of belonging and complicating socio-emotional relationships. This review further underscores the importance of socio-emotional factors and their connection to the classroom social climate [28].

Students’ perception of the Classroom Social Climate (CSC) is critically important, particularly given the high rate of academic dropout among university students in Chile, reaching 24.4% in 2020 [29,30]. The national perspective on CSC remains unclear due to a lack of data regarding how this construct influences or relates to various academic variables in university settings. Therefore, this research is essential as an initial exploration, with one of the primary steps being the development of contextually appropriate instruments that demonstrate reliability and various validity indicators.

While CSC has been extensively studied among elementary and secondary school students, resulting in several tools designed for these levels, its exploration in university environments is less developed. Existing instruments are characterized by common factors such as the relationship between students and teachers, norms, and perceived social support [31,32,33,34,35,36]. However, there is a notable deficiency in a conceptual and operational framework for CSC at the university level [10], reflected in the scarcity of tools suitable for assessing this construct in higher education settings. Recognizing the differences between the educational contexts of primary and secondary education and higher education is essential [15,37,38,39].

The University Classroom Social Climate Scale (in Spanish, ECSA-U [9]) is a prominent tool among the instruments designed to assess this construct. Notably, the UCSCS was developed by adapting the Compulsory Secondary Education Classroom Social Climate Scale (ECSA-ESO) created by Pérez, Ramos, and López [40]. The latter scale comprises 41 items that evaluate nine dimensions of CSC among secondary students: (1) Interest/Respect/Concern, (2) Satisfaction/Expectations, (3) Relationship, (4) Competitiveness/Favoritism, (5) Communication, (6) Cooperation/Democracy, (7) Norms/Discipline, (8) Group Cohesion, and (9) Physical Organization of the Classroom [40].

Later, Rostán et al. [10] adapted the ECSA-ESO to suit university settings better. This adaptation process in Spain involved a team of expert judges who assessed the content relevance of the original items. Following this evaluation, the items most applicable to university students were selected, linguistically tailored to this demographic, and analyzed through exploratory and confirmatory factor analyses.

As a result of the adaptation and validation process, the University Classroom Social Climate Scale (ECSA-U) identified three distinct factors of CSC in university settings: (1) relationships among students, (2) teacher performance and the teacher–student relationship, and (3) adherence to norms and a sense of belonging. The first factor concerns the behaviors and relationships students develop among themselves; the second encompasses teacher behaviors and their interactions with students; the third combines affective and normative components, stressing the importance of explicit operational norms within the classroom.

Substantial evidence supports the influence of the first two factors on students’ motivation and their adaptation to university life [41,42]. The classroom environment and the nature of interactions led by teachers significantly impact student motivation. Moreover, positive relationships within the classroom facilitate a smoother and more manageable adaptation process for students entering university life [43,44,45].

Other instruments measuring CSC include the Inventory of University Classrooms (IACU [46]). The IACU assesses CSC across seven dimensions and offers two forms: one evaluating the natural classroom environment and another the ideal environment, which may introduce response bias as participants are exposed to the items beforehand. Internal consistency was measured using Cronbach’s alpha coefficient, yielding acceptable but not optimal results for assessing CSC. The Educational Climate Survey for Universities (ECSA-U) exhibits superior psychometric properties with more robust reliability indices. The ECSA-U underwent a cultural adaptation process involving expert judges and exploratory and confirmatory factor analyses of its internal structure. It has also been adapted and applied in various cultural contexts within Latin America, contrasting with the IACU’s limited usage as reported in the literature.

On the other hand, the internal consistency of the Educational Climate Survey for Universities (ECSA-U) has been validated in studies conducted across European and Latin American contexts, particularly in Spain and Peru. The first study demonstrated good internal consistency with a Cronbach’s alpha of 0.84, confirming the reliability of its scores for measuring CSC [47]. The second study also reported a favorable reliability index for the scale at 84%; however, it employed the Kuder–Richardson formula [48]. This formula is less appropriate for ordinal data, underscoring the necessity for an adapted scale version that uses more suitable reliability indices, such as McDonald’s omega.

Classroom Social Climate (CSC) is a high-impact phenomenon within the educational context; however, research on CSC in higher education remains limited, especially in Latin America. This highlights the need for valid and reliable tools to accurately measure CSC within universities accurately. Additionally, CSC is closely linked to motivation, influencing how students think, feel, and behave throughout their learning processes. This study aimed to analyze the psychometric properties of the University Classroom Social Climate Scale (ECSA-U, in Spanish) among Chilean university students from the La Araucanía region. The general objective encompassed four specific objectives: (a) to culturally and semantically adapt the ECSA-U to the Chilean context; (b) to determine the factorial structure of the ECSA-U; (c) to estimate the internal consistency as a measure of reliability of the ECSA-U; (d) to obtain evidence of convergent validity through the relationship of the instrument with the motivation scale from the Motivation and Learning Strategies Questionnaire (CMEA). Regarding the latter objective, it was hypothesized that there would be a significant and positive correlation between the ECSA-U and the motivation subscale of the CMEA.

## 2. Materials and Methods

### 2.1. Participants

The design of this study is quantitative, non-experimental, and cross-sectional. A total of 422 Chilean university students were recruited (between September and November 2023) through a stratified non-probabilistic sampling. Considering the nature of the variable to be measured, two aspects were estimated for the stratification: the first was the faculty according to the diversity of careers within the university, and the second was the students’ semester, given that the process of university life changes the students’ perceptions over time.

Based on the variables, it is adjusted to the parameters proposed by Kline [49], where it is suggested that for each instrument item, ten people are recruited; considering that the ECSA-U has 22 items, a minimum of 220 participants was required. On the other hand, the recommendations of the International Test Commission [50] mention that those studies that evaluate the factorial structure of an instrument should have more than 300 participants. This was complemented with an a priori test power analysis through the SEM Power package [51] for R Studio ver. 2023.06.0 using as parameters the probability of obtaining an RMSEA lower than 0.08, a statistical power of 0.80, and a nominal alpha of 0.05, obtaining a minimum sample of 130 participants. Therefore, the total sample exceeds the existing recommendations.

### 2.2. Measures

#### 2.2.1. Ad Hoc Sociodemographic Questionnaire

The survey assessed age, gender, faculty, year of study, course, semester, and subjective perception of class attendance.

#### 2.2.2. Classroom Social Climate

The University Classroom Social Climate Scale (ECSA-U [9]) was used. This instrument was developed in Spain, consisting of 22 items that assess the CSC in terms of 3 factors: Relationships among students (9 items), Teacher performance and teacher–student relationship (7 items), and following the rules and feeling of belonging (6 items). The response format is a four-point Likert-type from 1 = “Never” to 4 = “always”, and consists of items such as “We respect and show interest in all classmates”. The scale has an excellent internal consistency (Cronbach’s alpha = 0.84).

#### 2.2.3. Motivation 

The motivation subscale of the Motivation and Learning Strategies Questionnaire (CMEA) was used. This questionnaire was designed by Pintrich [52] to evaluate university students’ motivation and use of learning strategies. The questionnaire comprises 81 items in two sections; only the motivation scale (31 items) was applied [53]. The questionnaire items have a 7-point Likert-type response format, from 1 = “Strongly Disagree” to 7 = “Strongly Agree”, and the motivation subscale consists of items such as “I feel my heart racing when I give a test”. In addition, it has been validated in the Chilean population with an acceptable internal consistency (Cronbach’s alpha = 0.87), which makes it an adequate instrument to measure the construct [53].

### 2.3. Procedure

Although Spanish speakers originally designed the scale and it did not require translation, a linguistic adaptation of the items was necessary to tailor it to the Chilean context. To this end, a version of the University Classroom Social Climate Scale (ECSA-U) was developed to measure social climate perception in Chilean university classrooms. This adapted version underwent a committee review to ensure semantic equivalence with the original scale. Subsequently, the revised version was evaluated by a team of expert judges. These steps were integral to the cultural and semantic adaptation of the instrument.

Subsequently, a digital pilot test of the scale was conducted using the QuestionPro^®^ platform (https://www.questionpro.com/es/, accessed on 6 May 2023). At the end of the scale, a section was included to gather participants’ feedback on various aspects, such as the form and comprehension of the items. This feedback further refined the instrument’s suitability, characteristics, comprehension, form, and usability.

Finally, the adapted version of the instrument was obtained, and students were invited to participate through e-mails, posters with QR codes placed on strategic murals, and invitations in person at various places in the university, where the purpose of the study was explained in summary form. Those who agreed to participate completed both the informed consent form and the instruments virtually through the QuestionPro^®^ platform, and the time required to answer both instruments was between 10 and 15 min.

Regarding ethical aspects, the research protocol was approved by the Ethical Review Board of the Universidad de La Frontera through Act No. 069/23, using the Ethical Principles of Psychologists and Code of Conduct [54], all this to establish the necessary measures to ensure the protection of the participants, specifically the confidentiality of the responses, since the data analysis was performed from the data conglomerate and not individually. The voluntary nature of participation was ensured through the signing of the informed consent form, in addition to the possibility of leaving the study at the time the participant deems it convenient without any consequence for this. This ensured that all the information collected was strictly confidential, ensured the anonymity of the participants, and was only used for the study. 

### 2.4. Statistical Analysis 

Descriptive analyses were conducted to characterize the sample, and an exploratory structural equation modeling (ESEM) was performed to assess the instrument’s factor structure. The ESEM approach integrates the strengths of both Exploratory Factor Analysis (EFA) and Confirmatory Factor Analysis (CFA), allowing for a more flexible and robust assessment of the latent structure within data. While EFA is primarily utilized to explore underlying relationships among variables without imposing prior constraints, CFA focuses on validating a specific theoretical model, examining how observed variables relate to predefined latent factors. ESEM, on the other hand, enables researchers to simultaneously model multiple latent factors and their interrelationships, facilitating the identification of complex structures in the data and evaluating how variables may load onto more than one factor [55]. This approach is beneficial in contexts where relationships among variables are expected to be non-unidimensional. The Weighted Least Squares Mean and Variance adjusted (WLSMV) estimator was used, following guidelines for ordinal data [56]. Factor loadings (λ) of 0.300 or higher were acceptable [57].

The model fit to the data was evaluated with Chi-Square, Comparative Fit Index (CFI), Tucker–Lewis Index (TLI), Standardized Root Mean Residuals (SRMR), Root Mean Squared Error of Approximation (RMSEA) with its 90% interval. These indices were interpreted according to conventional goodness-of-fit criteria: CFI and TLI > 0.95 and SRMR and RMSEA < 0.08 [58,59,60]. After confirming the instrument’s factor structure, reliability analysis was conducted using McDonald’s omega coefficient (ω) and Greatest Lower Bound (GLB) recommended for multidimensional structures [61]. A coefficient above 0.70 is considered adequate for demonstrating satisfactory internal consistency [62].

For convergent validity, associations between variables were identified using Spearman’s correlations. Descriptive and bivariate statistics were performed using the JASP software v0.18.1, while multivariate analysis was conducted using Mplus v8.2. Statistical decisions were made with a nominal alpha ≤ 0.05.

## 3. Results

### 3.1. Descriptive Analyses

Regarding the characteristics of the sample, the average age of participants was 20.7 years (SD = 2.075). The gender distribution was as follows: 51.4% of participants identified as female, 46.6% as male, 0.2% as transgender, 1.2% as non-binary, and 0.4% preferred not to respond or selected ‘other’. The distribution by faculty was 18.7% from the Faculty of Agricultural and Environmental Sciences, 18.4% from the Faculty of Legal and Business Sciences, 22.0% from the Faculty of Engineering and Sciences, 18.4% from the Faculty of Education, Social Sciences, and Humanities, 18.2% from the Faculty of Medicine, and 4.02% from the Faculty of Dentistry.

### 3.2. Cultural and Semantic Adaptation

Initially, a proposal for the ECSA-U was developed to refine the semantic details of the scale, such as changing the term ‘class-group’ to ‘class’ and ensuring the inclusivity of the items by adding ‘he/she’ where necessary, among other adjustments. This proposal was reviewed by a committee of three professionals who were solely involved in this stage and did not participate as expert judges. An example of an item with these modifications is included. Subsequently, a team of four expert judges was assembled to assess the content validity of the ECSA-U proposal and to provide recommendations for enhancing the phrasing or content of the items. The methodology proposed by Escobar-Pérez and Cuervo-Martínez [63] was employed, utilizing two templates: one for recording the expert judge’s details and another for categorizing and annotating each item based on four criteria: clarity, coherence, relevance, and sufficiency [64]. This review was conducted individually, with each expert judge scoring each item in the proposal from one to four, except for sufficiency, which was evaluated once per dimension/factor. The expert team included a translator experienced in linguistics and semantics, along with three university professors who specialized in measurement, psychometrics, and education.

After collecting the comments and ratings from the expert judges, the concordance of their evaluations was estimated using Aiken’s V coefficient. This measure quantifies content validity based on a specific characteristic [65], considering the assessments of a panel of expert judges on an item or group of items [66]. The results indicated a high degree of concordance among the expert judges across all evaluated categories: Clarity = 0.91, Coherence = 0.87, Relevance = 0.88, and Sufficiency = 0.81. Although the sufficiency indicator was high for all the factors of the scale, the team of expert judges suggested incorporating items into factor 3 (Follow-up of norms and sense of belonging) based on comments such as “It is necessary to identify or highlight whether the students consider that the norms are effectively complied with”; five items were incorporated into this factor, as detailed in Table 1.

Once this version was obtained, a pilot survey was conducted virtually through the QuestionPro^®^ platform, and the objective of the pilot was to receive feedback from the participants regarding the characteristics of the scale. The pilot was answered by five psychology students at the Universidad de La Frontera. They stated that the scale was understandable, accessible, and easy to answer and that they needed only an average of 10 min to answer it. Linguistically, the participants mentioned that the items were relevant and representative to address the construct. They did not provide direct comments regarding any specific item or modifications needed.

### 3.3. Construct Validity Based on Factor Structure

An ESEM was configured with indicators grouped into their respective factors, allowing them to freely load onto other factors to evaluate cross-loadings or representational issues. This model fits the data well (CFI = 0.978, TLI = 0.971; RMSEA = 0.058 [90% CI 0.052–0.063], SRMR = 0.029). The factor loadings for fixed dimensions and freely estimated for other dimensions are presented in Table 2.

Upon inspecting the results, it was observed that three items (items 2, 15, and 16) exhibited cross-loadings and, therefore, were not exclusively representative of their original factors, leading to their removal from the scale. Additionally, another set of items (6, 7, 17, and 19) showed significant loadings on factors different from those originally designated. In the re-specified model, these items were reassigned based on their theoretical congruence.

### 3.4. Internal Consistency

An internal consistency analysis was conducted for the factors comprising the factor model using McDonald’s omega and GLB. The results show that all three factors have values above 0.7, indicating good reliability indices (see Table 3).

### 3.5. Correlation Analysis

A correlation test was conducted to obtain evidence of convergent validity between the results obtained on the ECSA-UCL and the Motivation subscale. Spearman’s rho test was used since the normality assumption was not met (*p* < 0.05). The results show a statistically significant correlation between the Motivation subscale and each of the factors of the ECSA-UCL; the correlation for each factor was positive and ranged from moderate to large in magnitude (see Table 4). This indicates that higher perceptions of Classroom Social Climate are associated with greater motivation.

## 4. Discussion

This study aimed to analyze the psychometric properties of the University Classroom Social Climate Scale (ECSA-U) in a sample of Chilean university students from the Araucanía region. Based on this, psychometric evidence of the scale’s internal structure in the national population was obtained, and the model proposed by Rostan et al. [10] was partially confirmed. Additionally, the scale demonstrated good reliability indices and provided evidence of convergent validity with the Motivation subscale and content validity.

As a result of the entire process, an adapted version of the ECSA-U, named ECSA-UCL, was developed. Unlike the original, this version includes 24 items, as five new items were added to the Norms Compliance and Sense of Belonging factor following the semantic adaptation process and suggestions from the expert judges. It is important to note that this process provided content validity evidence for the ECSA-UCL and demonstrated the suitability of the items that made up the original scale. Furthermore, these new items were incorporated into factor 3 to approximate the construct better.

Regarding the results obtained, three items (2, 15, and 16) were removed from the scale because they did not uniquely represent their original factors and were associated with another latent variable of the scale. This phenomenon can occur when a scale undergoes a validation process [67]. In this case, it may be that these items explore joint processes that do not necessarily co-occur. For instance, item 2 refers to respecting and showing interest; respect may be subject to the general norms of the classroom, addressing a more structural aspect of classroom functioning, while the display of interest would reflect the quality of relationships among peers. Item 16 again mentions two processes: one of communication and another related to managing norms to achieve compliance. Therefore, the first element alludes to a more structural component of norm communication, while the second pertains to teacher behavior. Both processes are captured separately and specifically, with items 23 and 26 added to the third factor. In the case of item 15, being non-specific about the figure that promotes discussions, it can capture variance from both the first factor (student relationships) and the second (teacher performance).

Additionally, three items were reassigned to different dimensions due to their theoretical congruence, reflecting the sociocultural context and the adaptation process’s consequences [68,69]. These items were 6, 7, 17, and 19, all originally belonging to the factor of Norms Compliance and addressing the latter. Items 7 and 19 refer to aspects of a sense of belonging that, in this analysis, were not related to the elements of norm adherence; instead, they loaded onto the peer relationship factor. This finding is consistent, as a sense of belonging is strongly linked to the quality of relationships, especially among peers. In the case of item 6, the feeling of comfort in the course is explicitly addressed, which is in a university environment where various subjects are taught, which is primarily influenced by the characteristics of the instructor. A similar situation occurs with item 17, which focuses on the instructor’s role in creating a pleasant learning climate; therefore, it would ultimately be congruent for it to represent factor 2.

Regarding the norm’s compliance factor, it should be noted that it underwent the most necessary modifications to address its goal better. The literature also evidences that explicit norms positively contribute to a good perception of CSC [70]. Overall, the three-factor structure is consistent with the literature [71]. All dimensions achieved excellent indices of internal consistency, calculated using robust estimators for cases where the factorial structure is multidimensional [61]

Given the limited number of studies focusing on the perception of CSC among university students at the national and Latin American levels, this study is an important precedent that highlights processes often overlooked in the development of university students, and that can be related to how they adapt to university life [72]. Moreover, the evidence of convergent validity obtained through the Motivation subscale demonstrates how the perception of CSC can also be related to students’ motivation. Additionally, this motivation can be linked to phenomena such as persistence or academic dropout, as discussed in the literature [73].

### Limitations and Future Research

This study has strengths and limitations. Among its strengths, the methodological approach used is notable, as it rigorously describes the approach to the construct, the process through which the research was conducted, and the proposed analyses. On the other hand, as a limitation, the study’s cross-sectional design can be mentioned since it limits understanding of the temporal stability of the CSC, and the scale is self-reported, which may involve some bias from the participants. As a self-report instrument, it is not free from social desirability biases [74]. However, self-reporting remains one of educational research’s most used and validated methods to capture students’ experiences and perceptions. Therefore, it is essential to ensure the scales’ psychometric quality, which was this study’s central objective. Regarding the sample, while it was obtained from a region characterized by multiculturalism and from an institution that receives students from various regions of Chile, it was collected using a non-probabilistic method. Consequently, the generalization of results should be cautiously approached, and future studies may conduct confirmatory analyses of the proposed factorial structure with larger samples that include other regions of the country. Future research should consider factors such as academic performance, stress, and the potential role of motivation in this pattern of relationships. Additionally, conducting a longitudinal study that tracks fluctuations in the perception of CSC over an academic year would be beneficial.

## 5. Conclusions

This study’s general objective was to analyze the psychometric properties of the ECSA-U in Chilean university students. Regarding the specific objectives, first, the cultural and linguistic adaptation carried out through expert judges and the pilot test resulted in a scale adjusted to the context of the application, in which relevant items were incorporated to understand the phenomenon of classroom social climate in university students and to capture more specifically the dimension of adherence to norms. These items reflect the students’ perspectives on communicating norms, conflict management, and the corresponding sanctions. Making these adaptations to the scale allows for minimizing biases due to the participants’ understanding and, secondly, ensures the satisfactory capture of relevant aspects of the phenomenon within the context in which it is applied, especially considering that research in this population segment is scarce.

The second specific objective was related to determining the factorial structure of the scale; in this regard, evidence was obtained that partially supports the dimensions evidenced by previous authors. During the analysis, it was identified that some items were ambiguous since they pointed to two scale dimensions and not exclusively to the one they corresponded to in the original version. On the other hand, other items loaded on dimensions other than those initially proposed. The result was a modified scale that demonstrated theoretical and statistical consistency. This is beneficial for the instrument’s validity since having dimensions that present greater theoretical clarity makes it easier for future studies to delve into more specific hypotheses, using some of the dimensions if they deem it necessary. In addition, the fact that the factorial structure was confirmed and that the incorporated items loaded on the corresponding dimension proves that these are relevant elements for measuring the norm-following dimension.

A third objective was determining the scale’s reliability through internal consistency. Excellent indicators were found in this regard, indicating that the dimensions consistently measure the construct. This is highly relevant in the scientific study of classroom social climate since it allows us to be sure of the accuracy of the measurement, especially considering that some antecedents of the scale had not opted for sufficiently robust estimators to determine this psychometric property.

Finally, the fourth objective aimed to determine the scale’s convergent validity with a measure of motivation. The results showed that the dimensions of the ECSA-UCL are related to the motivation measure. This result is relevant at the psychometric level since it provides evidence of validity to the scale, considering that the literature points out that, indeed, a better classroom social climate is linked to higher levels of motivation, the latter being a central factor for academic success and well-being within educational environments.

Although this is the first approximation of the construct specifically for university students, having this instrument makes it possible to continue working on measuring the perception of CSC in university students so that its usefulness can be diversified. It can be used to evaluate CSC before and after an intervention, even contributing to monitoring strategies to improve the quality of education in an educational institution.

## Figures and Tables

**Table 1:** Items added to factor 3.

Items
-The professors have clearly informed us about the program regulations and how they function. -*Los/as docentes nos han informado claramente sobre el reglamento de la carrera y su funcionamiento*.
-The university protocols and how they are executed have been explained to us as students. -*A los/as estudiantes se nos han explicado los protocolos de la universidad y la forma en que se ejecutan.*
-The professors have clearly informed us about the norms and rules of this course. *-Los/as docentes nos han informado claramente sobre las normas y reglas de este curso.*
-We, as students, believe that violations or breaches of the regulations are appropriately sanctioned. *-Los/as estudiantes consideramos que las denuncias o faltas a los reglamentos son debidamente sancionadas.*
-We, as students, believe that our complaints are heard and addressed by the program director. *-Los/as estudiantes consideramos que nuestras quejas son escuchadas y atendidas por el/la directora/a de carrera.*

**Table 2:** Factor loading for ESEM analysis.

Item	F1	F2	F3
1.- El/la docente se preocupa de resolver las dudas que planteamos durante la clase. *The teacher is concerned about addressing the questions we raise during class.*	0.014	**0.860**	0.069
2.- Respetamos y mostramos interés por todos/as los/as compañeros/as del curso. *We respect and show interest in all classmates in the course.*	0.380	0.372	0.025
3.- El/la docente promueve el respeto por las creencias, ideas y sentimientos de todos y todas las participantes del curso. *The teacher promotes respect for the beliefs, ideas, and feelings of all participants in the course.*	0.092	**0.752**	0.108
4.- El/la docente se muestra satisfecho/a cuando logramos el aprendizaje de competencias profesionales. *The teacher is satisfied when we learn professional skills.*	0.152	**0.804**	0.020
5.- El/la docente promueve la ayuda y cooperación entre nosotros/as. *The teacher promotes help and cooperation among us.*	0.128	**0.692**	0.155
6.- Los/as estudiantes nos sentimos cómodos/as en este curso. *The students feel comfortable in this course.*	0.129	**0.849**	0.038
7.- Como estudiante, me siento orgulloso/sa de pertenecer a mi carrera. *As a student, I feel proud to belong to my program.*	**0.623**	0.036	−0.087
8.- El/la docente logra que los/as estudiantes nos sintamos motivados para asistir a este curso. *The teacher makes students feel motivated to attend this course.*	0.081	**0.755**	0.170
9.- La relación entre el/la docente de esta clase y los/as estudiantes suele ser cordial. *The relationship between the teacher and the students in this class is usually cordial.*	0.125	**0.761**	0.083
10.- En nuestro curso, los/as estudiantes tenemos una buena relación entre nosotros/as. *In our course, the students have a good relationship with each other.*	**0.811**	−0.098	−0.032
11.- En nuestro curso, los/as estudiantes prestamos atención a las intervenciones y aportes de nuestras compañeras y compañeros. *In our course, the students pay attention to the contributions of our peers.*	**0.797**	0.041	−0.029
12.- Los/as estudiantes actuamos para evitar situaciones de discriminación hacia otros/as compañeros/as. *The students take action to prevent discrimination towards other classmates.*	**0.632**	0.014	0.125
13.- Los/as estudiantes tenemos una buena comunicación con el/la docente de esta asignatura. *The students have good communication with the teacher in this subject.*	0.117	**0.742**	0.170
14.- Los/as estudiantes colaboramos entre nosotros/as. *The students collaborate with each other.*	**0.834**	−0.124	0.048
15.- En esta asignatura, se favorecen los debates entre nosotros. *In this subject, debates among us are encouraged*.	0.331	0.340	0.228
16.- El/la docente informa y hace cumplir las normas de comportamiento en el curso. *The teacher informs and enforces behavior rules in the course.*	0.110	**0.590**	0.311
17.- En esta clase, el/la docente y los/as estudiantes se preocupan por crear un clima propicio para el aprendizaje y el desarrollo académico. *In this class, the teacher and students strive to create a conducive climate for learning and academic development.*	0.198	**0.588**	0.254
18.- Los/as estudiantes de nuestro curso nos interesamos por formarnos como profesionales competentes. *The students in our course are interested in developing as competent professionals*.	**0.595**	0.017	0.164
19.- Mi carrera se imparte en un lugar agradable. *My program is taught in a pleasant place.*	**0.557**	−0.049	0.150
20.- Los/as estudiantes nos preocupamos del progreso de nuestros/as compañeros/as de curso. *The students are concerned about the progress of our classmates.*	**0.598**	−0.172	0.265
21.- A los/as estudiantes se nos ha informado claramente sobre el reglamento universitario y su funcionamiento. *The students have been clearly informed about the university regulations and how they work.*	−0.001	−0.093	**0.912**
22.- A los/as estudiantes se nos ha informado y explicado claramente las consecuencias del incumplimiento de las normas universitarias. *The students have been clearly informed and explained the consequences of non-compliance with university regulations.*	−0.094	−0.096	**0.961**
23.- Los/as docentes nos han informado claramente sobre el reglamento de la carrera y su funcionamiento. *The teachers have clearly informed us about the program regulations and their function.*	−0.083	−0.061	**0.962**
24.- A los/as estudiantes se nos han explicado los protocolos de la universidad y la forma en que se ejecutan. *The university protocols and how they are executed have been explained to us as students.*	0.015	−0.224	**0.933**
25.- Los/as docentes nos han informado claramente sobre las normas y reglas de este curso. *The teachers have clearly informed us about the norms and rules of this course.*	0.096	0.144	**0.672**
26.- Los/as estudiantes consideramos que las denuncias o faltas a los reglamentos son debidamente sancionadas. *The students believe that reports or violations of regulations are appropriately sanctioned.*	0.142	0.026	**0.543**
27.- Los/as estudiantes consideramos que nuestras quejas son escuchadas y atendidas por el/la director/a de carrera. *The students believe that our complaints are heard and addressed by the program director.*	0.169	0.181	**0.439**

Note: In bold, highest factor loading corresponding to the final dimension.

**Table 3:** Reliability analysis.

Factor	ω	GLB	Mean	SD
Relationships among students.	0.851	0.886	3.189	0.504
Teacher performance and teacher–student relationship.	0.947	0.960	3.055	0.747
Following the rules.	0.888	0.914	2.885	0.665

**Table 4:** Correlation analysis.

	Factor 1	Factor 2	Factor 3
Motivation	0.631 **	0.660 **	0.415 **

Note: ** *p* < 0.001.

## Data Availability

The raw data supporting the conclusions of this article will be made available by the authors on request.
